# Application of HPSEC Technique and In Silico Analysis in the Evaluation of Bioactive Peptides and Polysaccharide Profile in Wort Supplemented with Malted and Unmalted Hemp Seeds

**DOI:** 10.3390/molecules30183676

**Published:** 2025-09-10

**Authors:** Robert Duliński, Marek Zdaniewicz, Łukasz Byczyński, Krystyna Żuk-Gołaszewska, Bożena Bukowska

**Affiliations:** 1 Department of Biotechnology and General Food Technology, Faculty of Food Technology, University of Agriculture in Krakow, Balicka Street 122, 30-149 Krakow, Poland; lukasz.byczynski@urk.edu.pl; 2 Department of Fermentation Technology and Microbiology, Faculty of Food Technology, University of Agriculture in Krakow, Balicka Street 122, 30-149 Krakow, Poland; m.zdaniewicz@urk.edu.pl; 3 Department of Agrotechnology and Agribusiness, Faculty of Agriculture and Forestry, University of Warmia and Mazury in Olsztyn, Oczapowskiego Street 8, 10-719 Olsztyn, Poland; kzg@uwm.edu.pl; 4 Department of Biophysics of Environmental Pollution, Faculty of Biology and Environmental Protection, University of Lodz, Pomorska Street 141/143, 90-236 Lodz, Poland; bozena.bukowska@biol.uni.lodz.pl

**Keywords:** *Cannabis sativa* L., malted hemp, bioactive peptides, antioxidant activity, polysaccharides, prebiotic potential, HPSEC, functional beer

## Abstract

This study examined the profile of bioactive peptides and polysaccharides in beer wort enriched with malted and unmalted hemp seeds. The aim of this research was to evaluate the influence of different hemp processing methods (malted versus unmalted) on the concentration and characteristics of bioactive compounds—specifically (1) peptides exhibiting antioxidant, anti-inflammatory, and antihypertensive activities and (2) soluble polysaccharide fractions that affect wort viscosity and prebiotic potential. The results indicated that supplementation with 10% malted hemp seeds was most favorable. This level of addition enhanced the peptide composition of the wort without adversely affecting fermentation efficiency. Moreover, it facilitated the generation of functional peptides with antioxidant and flavor-enhancing properties and introduced non-fermentable polysaccharides that improved wort viscosity and foam stability without the negative effects observed at higher hemp seed concentrations. In contrast, a 30% addition of hemp seeds, particularly in unmalted form, led to a reduction in fermentable sugar and peptide contents and increased the likelihood of fermentation slowdown. The incorporation of 10% malted hemp seeds has the potential to enhance the sensory and functional attributes of beer, primarily due to the presence of bioactive peptides and polysaccharides, while maintaining fermentation performance and clarity. Fermentation and brewing efficiency may decline at higher hemp seed inclusion rates, warranting further investigation. The use of unmalted hemp necessitates enzymatic treatment to improve fermentable sugar availability. Additionally, high-performance size-exclusion chromatography (HPSEC) proved to be a valuable analytical tool for optimizing wort composition in the development of hemp-enriched beers.

## 1. Introduction

In recent years, the increasing demand for food ingredients with diverse functional properties has driven intensified scientific interest in alternative sources of plant-based proteins and bioactive compounds [1,2]. Hemp seeds are characterized by a distinctive chemical composition, featuring a complete profile involving essential amino acids, a high concentration of unsaturated fatty acids, and a rich array of polyphenols and cannabinoids. Extensive research conducted both in vitro and in vivo has revealed that these bioactive compounds possess significant antioxidant, immunomodulatory, vasorelaxant, neuroprotective, and antibacterial activities [3,4,5]. Seeds have traditionally been used as animal feed, but their processed products, such as oil, meal, flour, and protein powder, are gaining popularity on the market due to the growing interest in their use in the human diet [6,7]. The seeds contain oil (25–35%), protein (20–25%), and carbohydrates (20–30%), as well as small amounts of minerals such as phosphorus, potassium, magnesium, sulfur, calcium, iron, and zinc [8]. In addition, they have high antioxidant activity due to the presence of vitamins A, C, and E and beta-carotene [9,10]. Hemp seed oil, on the other hand, is rich in polyunsaturated fatty acids, which have industrial applications in cosmetic products, such as moisturizers and shampoos, massage oils, conditioners, and lotions [11], as well as very good health-promoting activity, and therefore show great potential as nutraceuticals [12].

Due to their favorable nutritional and functional properties, hemp seeds have been used in increasing applications in the food industry, including (1) baked goods, where up to 20% of wheat flour can be substituted with hemp flour [13]; (2) breads, including gluten-free varieties, in which starch is partially replaced with hemp flour or protein-enriched fractions [14]; (3) beverages, such as fermented hemp-based drinks [15] and hemp seed milk [16]; (4) energy bars, where up to approximately 40% of rice flour is replaced with defatted or whole hemp powder [17]; and (5) plant-based meat alternatives, where soy protein isolate is partially substituted with hemp protein concentrate [18].

Hemp seed proteins have garnered significant attention in both scientific research and the food industry due to their exceptional nutritional profile, high digestibility, and low allergenic potential [19,20]. Moreover, numerous studies have demonstrated that hydrolysates derived from hemp seed proteins exhibit a wide range of bioactivities, including antioxidant effects, antihypertensive activity through inhibition of renin and angiotensin-converting enzyme [21], antidiabetic effects via α-glucosidase inhibition [22,23], antitumor activity characterized by enhanced apoptosis and reduced viability and migration of Hep3B liver cancer cells [24], and immunomodulatory properties, evidenced by decreased expression of pro-inflammatory mediators (TNF-α, IL-1β, and IL-6) and increased levels of anti-inflammatory cytokines (IL-10 and IL-4) [25].

In silico protein digestion methods are used to predict the presence of specific bioactive peptides [26]. While in silico analysis offers a valuable, cost-effective, and rapid method for predicting potential bioactive peptides, it is important to acknowledge that these findings are theoretical. Peptide databases (e.g., BIOPEP-UWM) are extensive and offer predictive models based on known protein sequences and specific proteolytic activities [27]. Bioactivities identified in such studies (e.g., antioxidant, antihypertensive, and DPP-IV inhibitory effects) constitute potential activities and serve as a foundation for further in vitro and in vivo studies.

Hemp seeds also contain carbohydrates including about 27.6% fiber (5.4% soluble and 22.2% insoluble) [28]. Despite the high fiber content, the use of hemp seed polysaccharides (HSPs) in the food industry is limited due to the lack of complete characterization of their physicochemical properties [29].

Despite its many beneficial properties and the growing interest in hemp as a raw material across various industries, including the food and pharmaceutical sectors [5,7,30], its potential in the context of fermentation biotechnology and brewing wort production remains underexplored.

Industrial hemp is becoming an increasingly popular ingredient in beer production, especially in the craft beer sector [31]. The unique properties of hemp and its proteins and sugars can affect various aspects of beer quality, from foam stability to flavor and texture. Hemp proteins, especially those of medium molecular weight, can act as foam stabilizers in beer. Like barley proteins, they can participate in the formation and maintenance of foam, which affects the sensory perception of the beverage. However, too much of these proteins can lead to excessive viscosity or turbidity in this beverage.

While previous studies on the incorporation of hemp seeds into brewing substrates have predominantly addressed physicochemical properties, antioxidant capacity, and conventional brewing and sensory metrics [32,33,34], the molecular characterization of peptide and polysaccharide fractions remains underexplored.

To our knowledge, no investigation has yet combined high-performance size-exclusion chromatography (HPSEC) with in silico analysis to simultaneously assess both the molecular weight distribution and the predicted bioactivity of these fractions in worts supplemented with malted and unmalted hemp seeds. This integrated methodology provides detailed structural profiles of wort constituents and uncovers their potential functional roles, thereby filling a critical knowledge gap and introducing a novel analytical framework for brewing research.

## 2. Results and Discussion

### 2.1. Bioactive Peptides in Wort Depending on Grist Composition

#### 2.1.1. HPSEC Analysis

The variation in protein fractions in wort is a result of malt type, mashing parameters (temperature, time, and pH), and proteolytic enzyme activity. Their control allows for fermentation to be optimized, improving the sensory quality of beer and extending its shelf life and clarity reference. The molecular weight (MW) distribution of protein-derived compounds in wort is a critical factor influencing both the technological and nutritional properties of the final beer product.

The importance of different fractions of plant proteins in beer production is well known, but there is a lack of sufficient knowledge regarding the role of hemp proteins.

**Figure 1:**
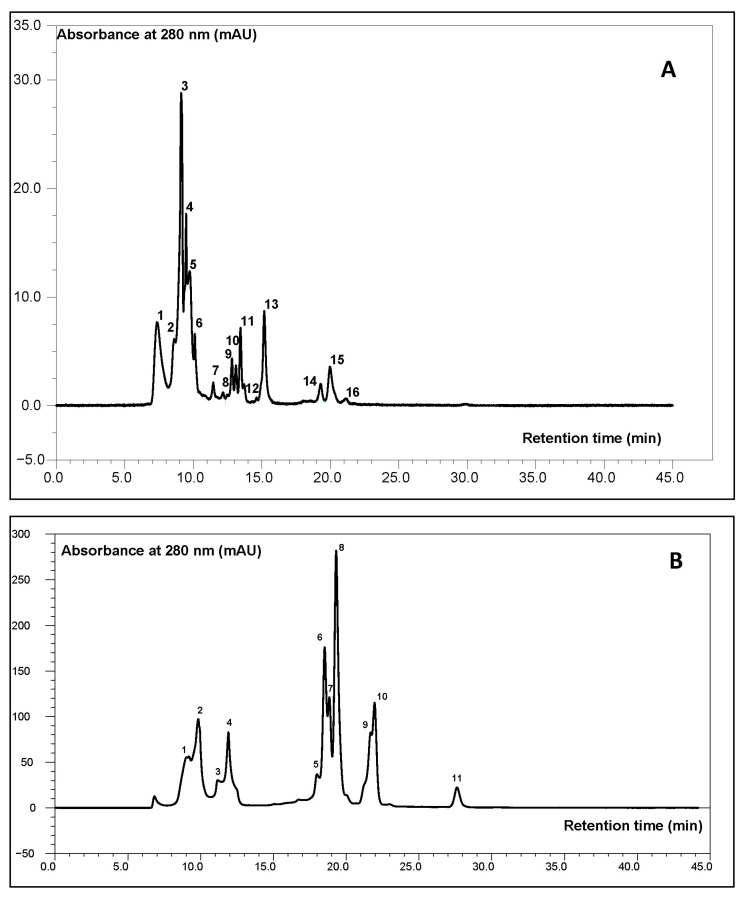
Sample chromatograms from HPSEC analysis of the polypeptide fractions of hemp seeds (**A**) and barley wort with the addition of 10% malted hemp seeds (**B**) on TSK-Gel G2000SWXl column.

**Figure 2:**
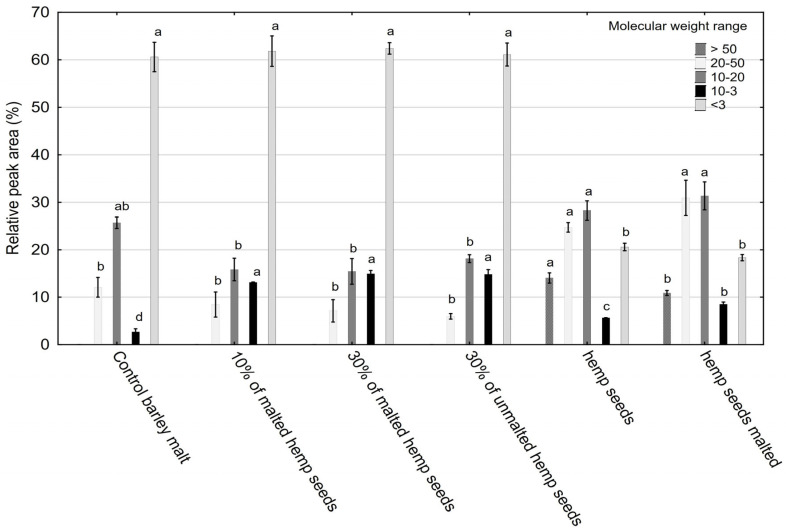
Molecular weight distribution of polypeptide fractions analyzed using TSK-Gel G2000SWXL SEC-HPLC column. One-way analysis of variance and Tukey’s post hoc test were applied. Mean values (n = 4) within bars followed by different letters differ significantly (*p* ≤ 0.05).

Based on the HPSEC results (Figure 1), a clear differentiation in the profile of protein fractions was observed depending on the type and amount of hemp seed additive used. Low-molecular-weight peptides (<3 kDa) predominated across all samples, particularly in those supplemented with 10% malted hemp seeds (69.2%) and 30% malted hemp seeds (62.5%) (Figure 2). These peptides are important for their bioavailability, potential antioxidant and antihypertensive activities, and contribution to Maillard reactivity. The control sample also showed high levels (59.5%), attributable to barley malt proteolysis. Hemp seeds and malt were found to contain very low amounts of peptides with molecular weights below 3 kDa, whereas their presence in the tested systems was primarily derived from barley malt, as confirmed by statistically significant differences (*p* = 0.000135). However, no significant differences were observed between barley malt and malts supplemented with hemp, indicating that the addition of hemp does not disrupt the fractional structure of polypeptides.

In addition, plant protein fractions of medium molecular weight, primarily glycoproteins, play a crucial role in the formation and stability of beer foam viscosity and body. Their hydrophobicity and appropriate molecular weight are key factors influencing foam stability. In turn, excessive protein hydrolysis can reduce the levels of these fractions, resulting in diminished foam quality [35,36]. High-molecular-weight proteins, particularly those that form complexes with polyphenols, contribute to cold turbidity [37]. The presence of these proteins must be carefully controlled, for example, through the use of bentonite, polyvinylpolypyrrolidone (PVPP), or optimized filtration conditions [38].

Mid-range peptides (10–50 kDa) were more abundant in worts enriched with 30% malted (14.96–15.66%) or unmalted (11.0–22.3%) hemp seeds (Figure 2), suggesting edestin-derived bioactive peptides. Hemp seeds are notable for their high content of quality proteins, such as edestin and albumin, which significantly enhance their nutritional value [39].

The levels of mid-range and high-molecular-weight peptides (~30%) in hemp seeds and malt were significantly higher than in all other probes (*p* = 0.000135). The lowest levels of high-MW peptides were observed in barley malt samples with 10% and 30% additions of malted hemp seeds and 30% unmalted hemp seeds. The high contents of mid-range and high-molecular-weight peptides in hemp seeds and malt suggest the presence of large proteins and their degradation products. These compounds can negatively affect beer quality if not properly broken down during brewing. However, the addition of hemp seeds (10% and 30%, malted and unmalted) to barley malt significantly reduced the high-MW fraction to levels lower than in pure barley malt. This indicates that malting and/or proteolytic enzyme activity effectively degrades hemp-derived high-MW proteins, which are associated with chill haze and reduced beer clarity. Thus, decreasing the levels of these fractions can be considered advantageous. Minimal presence of high-MW fractions (>50 kDa) across all samples indicates efficient proteolysis during mashing. These results support the idea that hemp seed supplementation modulates protein degradation pathways during mashing, potentially enhancing the release of bioactive peptides.

The observed decrease in low-molecular-weight peptides in the malted hemp wort may have a negative impact on foam stability, as these fractions are known to be critical for the formation of stable foam-active proteins. Conversely, the presence of larger peptides could contribute to a fuller mouthfeel. Peptides can also act as precursors for aroma compounds during fermentation and storage. The identified specific peptide profile may lead to innovative flavor profiles in the final beer, but this requires additional sensory testing on the final product.

Additionally, peptides and amino acids serve as vital nitrogen sources for yeast during fermentation. Insufficient levels of free amino nitrogen (FAN) can lead to sluggish fermentation and the production of undesirable metabolites, such as higher alcohols and hydrogen sulfide [40,41]. Also, certain proteins and their fragments (e.g., prolamines) can affect the mouthfeel, viscosity, and body of beer. Too few protein fractions result in “watery” beers and too many cause excessive viscosity or turbidity [42,43]. Small peptides, on the other hand, can participate in Maillard reactions with reducing polysaccharides during wort boiling, which can affect color, flavor (e.g., caramel and toast), and oxidative stability [44,45].

#### 2.1.2. In Silico Analyses

Based on the in silico analysis using subtilisin A (EC 3.4.21.62) performed on selected barley (*Hordeum vulgare*) and hemp (*Cannabis sativa*) proteins, it can be concluded that both groups of proteins are a rich source of bioactive peptides (Table 1). For barley proteins, the theoretical degree of hydrolysis (DHt) ranged from 20.19% for C-hordein (P06472) to 26.03% for B1-hordein (P06470). The hydrolysis of B1-hordein resulted in the release of peptides with 13 different activities, with the highest number of peptides having dipeptidyl peptidase IV (DPP-IV) inhibitory activity. C-hordein, on the other hand, yielded peptides with 4 types of bioactivity, while D-hordein (I6SW23) yielded peptides with 13 different activities, with the largest number of them having DPP-IV inhibitory properties. Hemp proteins were characterized by a higher degree of hydrolysis, ranging from 27.14% for 11S seed storage protein (A0A090CXP9) to 34.58% for the bifunctional protein (A0A803Q1B3). This suggests that the enzyme used in the study (subtilisin A) is more effective at breaking down hemp proteins, which would require experimental verification. The hydrolysis of 11S seed storage protein resulted in the formation of peptides with 17 different activities, with the highest number being inhibitors of DPP-IV and angiotensin-converting enzyme (ACE). Furthermore, peptides with activities such as calpain 1 inhibitors and neprilysin 2 inhibitors were released with very high efficiency (W = 1.0000) from both barley proteins (B1-hordein) and hemp proteins (11S seed storage protein, bifunctional protein, and storage protein).

In general, the dominant bioactivity among the released peptides from both barley and hemp was DPP-IV inhibitory activity, which indicates the great potential of these proteins as a source of bioactive peptides with a wide range of effects. The hydrolysis of hemp proteins led to the release of peptides with a greater variety of bioactivity types (e.g., 17 for 11S seed storage protein) compared to barley proteins (a maximum of 13 types). From hemp proteins, types of bioactivity that were not detected in barley proteins were also detected, such as HMG-CoA reductase inhibitors, antiviral, antidiabetic, and α-glucosidase inhibitors. Additionally, peptides with binding properties and glutamine carboxypeptidase II inhibitor were found in hemp proteins. However, among the peptides from barley proteins (B1-hordein), antiamnestic activity was detected, which was not noted in hemp proteins. Although both groups of proteins are a rich source of DPP-IV and ACE inhibitors, hemp proteins yielded a greater number of peptides with these activities. For example, 11S seed storage protein from hemp released 30 peptides with DPP-IV inhibitory activity and 16 peptides with ACE inhibitory activity, while B1-hordein from barley released 14 and 10 peptides, respectively. Furthermore, while both groups of proteins showed high W values (equal to 1.0) for some peptides (e.g., calpain 1 and neprilysin 2 inhibitors), hemp proteins showed this high degree of release for a greater number of different types of bioactive peptides (Table 2).

Peptides with activities such as ACE inhibitors, DPP-IV inhibitors, or antioxidants, which were identified in the study, can be used as functional ingredients in fermented beverages. ACE inhibitors have the potential to regulate blood pressure, while DPP-IV inhibitors may have applications in the treatment of type 2 diabetes. In turn, the discovery of various types of bioactivity, especially in hemp proteins (e.g., antiviral, antidiabetic, and α-glucosidase inhibitor activity), paves the way for the design of new nutraceutical products that can support health in various areas. Overall, this peptide prediction supports the functional enrichment potential of adding hemp seed to wort. Further experimental validation using LC-MS/MS is warranted to confirm the presence and stability of the predicted sequences during fermentation and storage. The analyses presented here constitute the basis for further experimental in vitro and in vivo studies aimed at confirming the biological activity of these peptides and assessing their potential use in fermented products and nutraceuticals.

### 2.2. Antioxidant Activity

**Figure 3:**
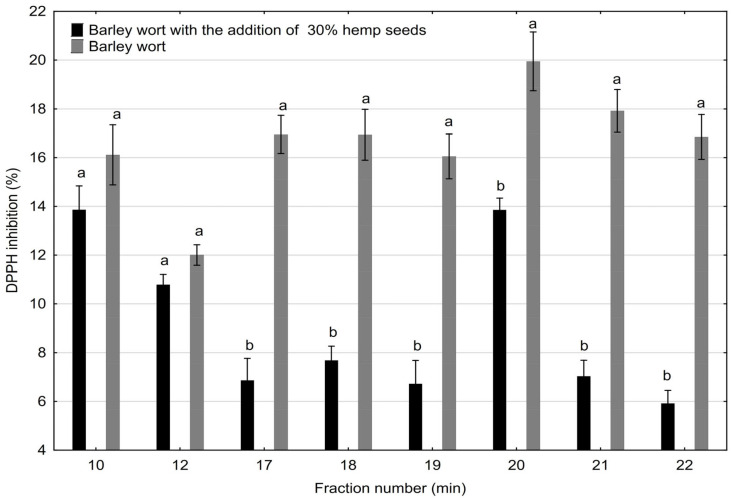
DPPH radical scavenging activity (%) of the selected polypeptide fractions of barley wort and barley wort with 30% malted hemp seeds separated using SEC-HPLC. The fraction number is directly correlated with the elution time in minutes on the HPSEC column. One-way analysis of variance and Tukey’s post hoc test were applied. Mean values (n = 3) within bars followed by different letters differ significantly (*p* ≤ 0.05) between respective fraction numbers.

There is a wide spectrum of tests used to analyze the antioxidant parameters of food products, and in this study, one of the most common tests was used, inhibiting the spectral properties of the DPPH radical (Figure 3).

Interestingly, slightly higher antioxidant activity was observed in the <50 kDa SEC-HPLC fractions (no. 17–22) derived from barley wort compared to those from wort supplemented with 30% malted hemp seeds. This effect can be attributed to several interrelated biochemical factors.

Firstly, the barley-derived peptides likely contain a higher proportion of amino acids with strong redox activity, such as histidine, tryptophan, tyrosine, and cysteine, which contribute significantly to radical scavenging potential [39,46]. Secondly, although hemp seeds are rich in phenolic compounds, these can form non-covalent or covalent complexes with proteins and peptides, potentially reducing their antioxidant effectiveness by masking reactive functional groups [36].

Moreover, the proteolytic processes that occur during hemp seed malting may generate peptides of smaller size but lower antioxidant capacity due to differences in sequence composition and structural accessibility. Finally, the partial substitution of barley malt with hemp may dilute the concentration of barley-specific antioxidative peptides, contributing to the observed differences in activity.

### 2.3. HPSEC Results—Polysaccharide Fractions

HSPs have a similar monosaccharide composition.

Hemp seeds contain more than 50 carbohydrates [29] which have a monosaccharide composition involving mannose, ribose, glucuronic acid, galacturonic acid, glucose, galactose, arabinose or fucose [47], supporting alcoholic fermentation. Fermentable sugars affect the “purity” and lightness of flavor. A smaller amount of them can result in a fuller, heavier flavor and lower alcohol content [29,47]. Hemp is high in polysaccharides, such as cellulose, xylans, pectin, xyloglucans and mannans [48], which are not fermented by yeast. High-molecular-weight polysaccharides derived from hemp may enhance wort viscosity, foam stability, and flavor fullness. The degradation of carbohydrates contributes to a reduction in wort viscosity [49]. Moreover, high-molecular-weight polysaccharides can exhibit prebiotic properties, which are particularly desirable in the development of functional beer [50]. However, excessive amounts of non-fermentable carbohydrates may inhibit fermentation efficiency and necessitate the use of exogenous enzymes, such as β-glucanase, to enhance polysaccharide breakdown [51].

Analysis using high-performance size exclusion chromatography (HPSEC) showed notable variations in the molecular weight profiles of saccharide fractions when comparing the control wort to samples supplemented with hemp-derived materials.

**Figure 4:**
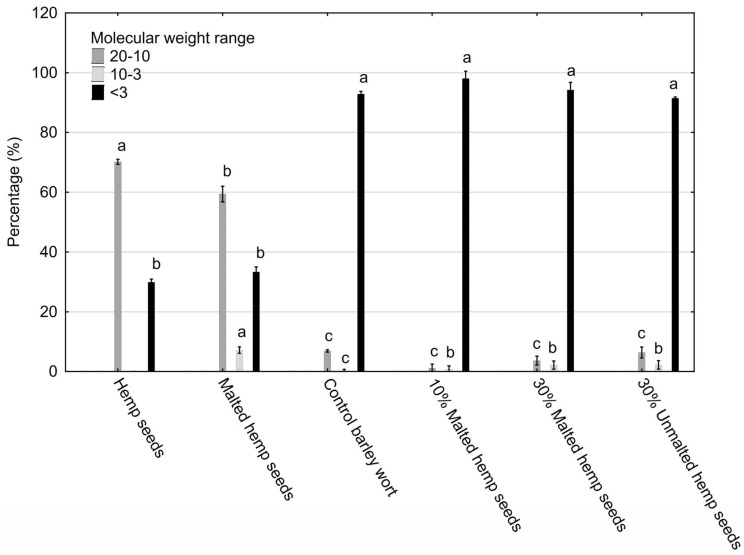
Molecular weight distribution of polysaccharide fractions analyzed on TSK-Gel G2000SWXL SEC-HPLC column. One-way analysis of variance and Tukey’s post hoc test were applied. Mean values (n = 4) within bars followed by different letters differ significantly (*p* ≤ 0.05).

In the control and 10–30% malted hemp seed samples, low-molecular-weight oligosaccharides (<3 kDa) predominated, constituting over 90% of the total saccharide content. These small molecules—primarily maltose, glucose, and low DP dextrins—are highly fermentable and contribute to efficient fermentation and a clean sensory profile in finished beer [51,52]. Hemp seeds and malted hemp seeds were shown to contain approximately half the amount of low-molecular-weight oligosaccharides compared to the other tested probes (Figure 4) with statistically significant differences (*p* = 0.00015).

Conversely, samples containing unmalted and malted hemp seeds exhibited a pronounced shift toward higher molecular weight fractions (10–50 kDa), indicative of non-starch polysaccharides such as arabinoxylans, cellulose, glucans, and galactomannans. These compounds are known for their functional properties, including improved beer foam stability, increased viscosity, and potential prebiotic effects in functional beer formulations [53]. Hemp seeds had the highest sugar content among all tested samples, with statistically significant differences (*p* = 0.00015) (Figure 4). Thus, hemp contains both fermentable monosaccharides (e.g., glucose and mannose) and large amounts of non-fermentable polysaccharides (e.g., cellulose, xylans, and pectin). Hemp seeds—both malted and unmalted—have significantly lower levels of fermentable sugars (<3 kDa) than barley malt, with reductions of up to 50% (*p* = 0.00015), limiting their contribution to alcoholic fermentation. Additionally, hemp seeds, especially when unmalted, contain the highest levels of high-molecular-weight polysaccharides (10–50 kDa), which improve viscosity, fullness of flavor, and foam stability; they can exhibit prebiotic effects but may hinder fermentation, particularly in the absence of enzymes capable of degrading these polysaccharides.

These findings indicate that high-molecular-weight saccharides derived from hemp may affect beer mouthfeel, fermentation kinetics, and biofunctional properties. However, to fully substantiate these effects, systematic investigations specifically targeting each of these parameters are required. For formulations involving unmalted hemp, exogenous enzymatic supplementation (e.g., β-glucanase and xylanase) may enhance fermentability.

Functional and Physicochemical Relevance:

- High-MW fractions (10–50 kDa): These enhance foam retention, colloidal stability, and mouthfeel and may bind bioactive compounds [54].
- Low-MW oligosaccharides (<3 kDa): These improve fermentability and alcohol yield and contribute mild sweetness and clean taste.

## 3. Materials and Methods

### 3.1. Materials

Hemp seeds of the Henola variety were used. The seeds were cultivated in Poland and delivered for research by Hemp Farm Poland (Krakow, Poland). Barley grains malted by Ireks (Kulmbach, Germany) were used as a reference. A detailed description of the ‘Henola’ cultivar is provided in our previous publication within the framework of this project [34].

### 3.2. Malting Procedure

The process of malting hemp seeds was carried out under laboratory conditions at the Department of Fermentation Technology and Microbiology of the University of Agriculture in Krakow according to our previous works [34,55]. The process consisted of three main stages: soaking the grain, germination, and drying. The total soaking time lasted six hours and consisted of nine water cycles and nine air cycles. For this purpose, 100 g of seeds was soaked in 500 mL of water at 19 °C for 30 min; then, the water was removed and the seeds were left out for five minutes while being constantly shaken. After completing the required number of soaking cycles, the samples were weighed and transferred for germination. During germination, the soaked seeds were exposed to a temperature of 20 °C. Every 24 h, their surfaces were sprinkled, and the entire batch was mixed. The germinated seeds were dried at 50 °C for 5 h to the desired moisture level (below 4% *m*/*m*).

### 3.3. Preparation of Laboratory Worts

#### Mashing

Three types of laboratory worts were produced using varying proportions of hemp seeds compared to a control wort made exclusively from barley malt. The tested variants, based on the methodology described by Zdaniewicz et al. [55], included 10% and 30% hemp malt and 30% unmalted hemp seeds. It was determined that the proportion of hemp seeds in the grist should not exceed 30% due to their low enzymatic activity and relatively low sugar content, which could significantly hinder efficient wort fermentation. All worts were prepared following the standard procedure for barley malt handling (EBC 4.5.1).

For this purpose, 50 g of malt milled in a laboratory grinder was weighed and placed into tarred mash containers, which were then placed in a water-heated apparatus at 45 °C. Agitators were set up, and the “Congress program” was selected. Next, 200 mL of distilled water at 45 °C was poured in portions into the containers. The apparatus was held at 45 °C for 30 min. Then, the temperature was raised at a rate of 1 °C/min until it reached 70 °C while constantly stirring the samples. When the apparatus reached 70 °C, 100 mL of distilled water warmed to the same temperature was added to the cups, and then the set temperature was maintained for 1 h. Next, the containers were cooled to 20 °C and filled to a mass of 450.0 g with distilled water, which was filtered through a paper filter. In order to ensure high clarity, the first portions of the filtrate were recirculated.

### 3.4. In Silico Analyses

Protein sequences were collected from the UniProt database, which was accessed on 1 August 2025 (https://www.uniprot.org/). The methodology for processing these sequences was similar to the one described by Minkiewicz et al. [56]. Protein UniProt accession numbers, names, lengths, masses, and sequences for the proteins are listed in Table 3 and Table 4. These sequences were then analyzed using the BIOPEP-UWM database, which is dedicated to bioactive and sensory peptides [26]. The BIOPEP-UWM database, provided by the University of Warmia and Mazury in Olsztyn, Poland, was accessed on 1 August 2025. This database generated a list of specific activities and locations of bioactive fragments, the frequency of these fragments, and the activities of fragments obtained through proteolysis.

Analysis of all protein sequences was performed using the following procedure: “Bioactive peptides → Analysis → Enzyme(s) Action → For your sequence → paste the protein sequence and choose enzyme → Report”. This method allowed for the calculation of parameters Ae, W, and DH (descriptions below). A complete detailed list of protein sequences and the frequency of all bioactive and sensory fragments is available in the BIOPEP-UWM protein database (https://biochemia.uwm.edu.pl/en/biopep-uwm-2/ (accessed on 1 August 2025)). The results from the BIOPEP-UWM analysis module provided the locations of bioactive fragments within protein chains and their predicted activity [27]. The frequencies of bioactive fragments in the protein sequence (A) were calculated according to Equation (1):

A = a/N (1)

where “a” is the number of fragments with a given activity in the analyzed protein sequence and “N” is the number of amino acid residues in the chain of this protein. The predicted frequency of release of bioactive fragments using proteolytic enzymes (Ae) was calculated according to Equation (2):

Ae = d/N(2)

where “d” is the number of peptides with a given activity (e.g., antioxidants) released as a result of the action of a specific enzyme or enzymes and “N” is the number of amino acid residues in the tested protein [57]. The relative frequency of release of fragments with given activity by selected enzymes was calculated according to Equation (3):

W = Ae/A(3)

where AE is the frequency of release of fragments with given activity by selected enzymes and “A” is the value previously described as the frequency of bioactive fragments’ occurrence in the protein sequence. The theoretical degree of hydrolysis (DHt) was calculated according to Equation (4):

DHt = d/D·100%(4)

where “d” is the number of hydrolyzed peptide bonds and “D” is the total number of peptide bonds in a protein chain.

Proteolysis simulations and quantitative parameter calculations for the proteins were performed using modules within the BIOPEP-UWM database. The proteolysis simulation of plant proteins was carried out using subtilisin A (EC 3.4.21.62). Bioactive fragments with data from the BIOPEP-UWM database [27] were then sought among the predicted proteolysis products.

### 3.5. SEC-HPLC Separation

Hemp seed proteins, malted hempseeds prepared according to the work of Zdaniewicz et al. [55], and wort samples were separated by SEC-HPLC on a Dionex Ultimate 3000 system (Thermo-Dionex, Sunnyvale, CA, USA). A volume of 100 µL of each sample (filtered through 0.45 µm membrane disks) was injected onto a TSK-Gel G2000SWXl (4 × 375 mm) semi-preparative column (Tosoh Bioscience, Tokyo, Japan). Subsequently, hemp seed isolate, malt, and wort samples were eluted from the column at a flow rate of 0.6 mL/min using an isocratic mode (solvent A: 0.01 M phosphate buffer, pH 7.00) for 45 min. Elution of polypeptide and polysaccharide fractions was monitored by absorbance at 280 nm and refractive index, respectively.

Molecular weight (MW) estimation was performed using a calibration curve constructed from the analysis of protein standards provided by Sigma-Aldrich (St. Louis, MO, USA, cat. no. 69385), including Ribonuclease A, Thyroglobulin, γ-Globulin, Ovalbumin, and *p*-Aminobenzoic acid, covering a molecular weight range of 15–600 kDa. The selection of this standard set was based on its broad MW coverage and proven suitability for calibrating HPSEC columns dedicated to peptide and protein fractionation. Calibration was further supported by external reference data from the column manufacturer (Tosoh Bioscience, Separation Report No. 46). Method validation included assessment of repeatability and reproducibility across three independent calibration runs. Each sample was analyzed in triplicate using biologically independent wort preparations (n = 3), and each chromatographic run was performed in technical duplicate to ensure consistency of retention times and reliability of peak area quantification.

Selected fractions of barley and barley with 30% malted hempseed wort samples were collected every minute using an automated fraction collector, model 2128 (Bio-Rad, Hercules, CA, USA), and pooled into fractions according to elution time. The pooled and combined fractions from four consecutive runs were evaporated using a Kamush evaporator (Lipopharm, Zblewo, Poland) and stored at −20 °C until required for further analysis. Each of the freeze-dried fractions was assayed for antioxidant activity using the DPPH scavenging test.

#### Preparation of Extracts for Antioxidant Activity Determination

For the analyses of antioxidant activity, 1 g of each sample was extracted with 80% aqueous ethanol (1:10, *w*/*v*) in the dark for four hours. The samples were then centrifuged at 4500× *g* (MPW-320 centrifuge; MPW Med Instruments, Warsaw, Poland) for 20 min at 4 °C. The supernatant was collected and used for total antioxidant capacity determination.

Antioxidant activity measurements were made using a methanolic (80%) DPPH solution at a concentration of 0.1 mmol/dm^3^. For the measurements, 0.2 mL of extracts and 2.8 mL of DPPH were used. Additionally, reference tests were performed using 0.2 mL of 50% acetone instead of a sample and Trolox (a synthetic derivative of vitamin E) equivalents. Samples were placed in the dark, and measurements were taken after 30 min at a wavelength of 516 nm using 80% methanol as a reference. Hydroxyl radical scavenging activity (%) was calculated using the formula ((A0 − A1)/A0) × 100, where A0 is the absorbance of the control and A1 is the absorbance of the sample.

### 3.6. Statistical Analysis

Experimental data were subjected to a one-way analysis of variance (ANOVA) to detect significant differences among means, and results are expressed as mean ± standard deviation (SD). Differences among means were checked by the Tukey or HSD test at *p* < 0.05 using Statistica for Windows, version 13.0 (StatSoft Inc., Tulsa, OK, USA), which is statistical software.

## 4. Summary and Conclusions

These results prove that incorporating 10% malted hemp seeds enhances the peptide and polysaccharide composition. This supplementation may positively affect antioxidant capacity, mouthfeel, and foam stability; however, these effects have yet to be evaluated in fermentation or sensory trials.

Higher hemp addition levels—particularly from unmalted seeds—produced less favorable wort profiles. The true impact of these additions on brewing performance must be confirmed in future studies.

The in silico analysis confirmed that both barley and hemp proteins are rich, theoretical sources of bioactive peptides. Our findings serve as a valuable guide for future research, suggesting specific areas for experimental validation using methods such as LC-MS/MS identification and in vitro assays to confirm the biological activity of the predicted peptides. This comprehensive approach would bridge the gap between computational prediction and confirmed biological function.

From this research, the following points can be summarized:

1. The addition of hemp—particularly at a 10% inclusion rate of malted seeds—may enrich wort with bioactive peptides and functional polysaccharides, potentially enhancing sensory attributes and health-related properties without compromising fermentation performance or clarity. While these results are promising, they must be validated through controlled fermentation trials and sensory evaluations before hemp can be credibly proposed as a novel raw material for craft and functional beer production.
2. Increasing the hemp content may alter the molecular structure of saccharides, which could in turn influence fermentability and sensory attributes.
3. The use of unmalted hemp requires enzymatic support to recover fermentable sugars.
4. HPSEC is a valuable tool for tailoring wort functionality in hemp-based beer development.

## Figures and Tables

**Table 1:** The results of in silico proteolysis with subtilisin (EC 3.4.21.62) of selected *Hordeum vulgare* (barley) proteins for the release of bioactive peptides.

Protein Code	DHt [%]	Type of Peptide Bioactivity	Number of Peptides	Ae	W
P06470	26.03	antiamnestic	1	0.0034	0.5000
		dipeptidyl peptidase IV inhibitor	14	0.0478	0.0593
		calpain 1 inhibitor	1	0.0034	1.0000
		Stimulating	3	0.0102	0.2991
		ACE inhibitor	10	0.0341	0.0568
		antioxidative	2	0.0068	0.0664
		dipeptidyl peptidase III inhibitor	1	0.0034	0.0433
		inhibitor of tripeptidyl peptidase II	3	0.0102	0.5965
		neprilysin 2 inhibitor	1	0.0034	1.0000
		xaa-pro inhibitor	1	0.0034	0.3333
		neuropeptide	1	0.0034	0.0907
		leucyltransferase inhibitor	1	0.0034	1.0000
		neprilysin inhibitor	1	0.0034	0.1245
P06472	20.19	ACE inhibitor	3	0.0286	0.0423
		dipeptidyl peptidase IV inhibitor	3	0.0286	0.0306
		xaa-pro inhibitor	4	0.0286	0.6008
		lactocepin inhibitor	3	0.0286	0.4288
I6SW23	24.40	dipeptidyl peptidase IV inhibitor	36	0.0482	0.0663
		inhibitor of tripeptidyl peptidase II	1	0.0013	0.0422
		ACE inhibitor	4	0.0054	0.0099
		antioxidative	2	0.0027	0.0255
		stimulating	1	0.0013	0.1215
		phospholipase A2 inhibitor	1	0.0013	0.4815
		renin inhibitor	1	0.0013	0.1083
		dipeptidyl peptidase III inhibitor	1	0.0013	0.0347
		neuropeptide	1	0.0013	0.0147
		anti-inflammatory	1	0.0013	0.1940
		xaa-pro inhibitor	1	0.0013	0.4815
		lactocepin inhibitor	1	0.0013	0.1625
		pancreatic lipase inhibitor	2	0.0027	0.3375
		hypotensive	1	0.0013	0.1215

**Table 2:** The result of in silico proteolysis with subtilisin (EC 3.4.21.62) of selected *Cannabis sativa* (hemp) proteins for the release of bioactive peptides.

Protein Code	DHt [%]	Type of Peptide Bioactivity	Number of Peptides	Ae	W
A0A090CXP9	27.14	calpain 1 inhibitor	2	0.0041	1.0000
		ACE inhibitor	16	0.0326	0.0711
		antioxidative	2	0.0041	0.0530
		stimulating	3	0.0061	0.1362
		dipeptidyl peptidase IV inhibitor	30	0.0611	0.0980
		dipeptidyl peptidase III inhibitor	4	0.0081	0.0864
		inhibitor of tripeptidyl peptidase II	4	0.0081	0.2207
		neprilysin 2 inhibitor	2	0.0041	1.0000
		HMG-CoA reductase inhibitor	1	0.002	0.4878
		leucyltransferase inhibitor	1	0.002	0.0578
		xaa-pro inhibitor	1	0.002	0.4878
		lactocepin inhibitor	1	0.002	0.1093
		acylaminoacyl peptidase inhibitor	1	0.002	0.4878
		tubulin-tyrosine ligase inhibitor	1	0.002	0.3279
		hypouricemic	1	0.002	0.0893
		pancreatic lipase inhibitor	1	0.002	0.2469
		antiviral	1	0.002	0.1399
A0A803Q1B3	34.58	neuropeptide	1	0.0047	1.0000
		dipeptidyl peptidase IV inhibitor	9	0.0419	0.0634
		calpain 1 inhibitor	1	0.0047	1.0000
		ACE inhibitor	4	0.0186	0.0400
		stimulating	2	0.0093	0.1332
		inhibitor of tripeptidyl peptidase II	1	0.0047	0.0842
		neprilysin 2 inhibitor	1	0.0047	1.0000
		antidiabetic	1	0.0047	0.5054
A0A7J6FEU0	31.16	calpain 1 inhibitor	1	0.0025	1.0000
		ACE inhibitor	15	0.0376	0.0725
		Antioxidative	3	0.0075	0.0623
		dipeptidyl peptidase IV inhibitor	24	0.0602	0.0961
		alpha-glucosidase inhibitor	1	0.0025	0.0767
		dipeptidyl peptidase III inhibitor	1	0.0025	0.0285
		anti inflammatory	1	0.0025	0.5000
		inhibitor of tripeptidyl peptidase II	2	0.005	0.1247
		neprilysin 2 inhibitor	1	0.0025	1.0000
		acylaminoacyl peptidase inhibitor	1	0.0025	1.0000
		tubulin-tyrosine ligase inhibitor	1	0.0025	0.1429
		glutamate carboxypeptidase II inhibitor	2	0.005	0.1247
		hypouricemic	1	0.0025	0.1244
		binding	1	0.0025	1.0000
		neuropeptide	1	0.0025	0.1244
		pancreatic lipase inhibitor	1	0.0025	0.2500

**Table 3:** List of selected Hordeum vulgare (barley) proteins used for bioactive peptide in silico analysis.

Protein Name or Function	Access. No in UniProt	Length (aa)	Mass (kDa)
B1-hordein	P06470 · HOR1_HORVU	293	33.422
Sequence: MKTFLIFALLAIAATSTIAQQQPFPQQPIPQQPQPYPQQPQPYPQQPFPPQQPFPQQPVPQQPQPYPQQPFPPQQPFPQQPPFWQQKPFPQQPPFGLQQPILSQQQPCTPQQTPLPQGQLYQTLLQLQIQYVHPSILQQLNPCKVFLQQQCSPVPVPQRIARSQMLQQSSCHVLQQQCCQQLPQIPEQFRHEAIRAIVYSIFLQEQPQQLVEGVSQPQQQLWPQQVGQCSFQQPQPQQVGQQQQVPQSAFLQPHQIAQLEATTSIALRTLPMMCSVNVPLYRILRGVGPSVGV			
C-hordein	P06472 · HOR7_HORVU	105	12.180
Sequence: QPQQSYPVQPQQPFPQPQPVPQQRPQQASPLQPQQPFPQGSEQIIPQQPFPLQPQPFPQQPQQPLPQPQQPFRQQAELIIPQQPQQPLPLQPHQPYTQQTIWSMV			
D-hordein	I6SW23 · I6SW23_HORVU	747	79.350
Sequence: MAKRLVLFVAVIVALVALTTAEREINGNNIFLDSRSRQLQCERELQESSLEACRRVVDQQLVGQLPWSTGLQMQCCQQLRDVSPECRPVALSQVVRQYEQQTEVPSKGGSFYPGGTAPPLQQGGWWGTSVKWYYPDQTSSQQSWQGQQGYHQSVTSSQQPGQGQQGSYPGSTFPQQPGQGQQPGQRQPWSYPSATFPQQPGQGQGQQGYYPGATSLLQPGQGQQGPYQSATSPQQPGQGQGQQETYPIATSPHQPGQWQQPGQGQQGYYPSVTSPQQSGQGQQGYPSTTSPQQSGQGQQLGQGQQPGQGQQGYPSATFPQQPGQWQQGSYPSTTSPQQSGQGQQGYNPSGTSTQQPGQVQQLGQGQQGYYPIATSPQQPGQGQQLGQGQQPGHGQQLVQGQQQGQGQQGHYPSMTSPHQTGQGQKGYYPSAISPQQSGQGQQGYQPSGASSQGSVQGACQHSTSSPQQQAQGCQASSPKQGLGSLYYPSGAYTQQKPGQGYNPGGTSPLHQQGGGFGGGLTTEQPQGGKQPFHCQQTTVSPHQGQQTTVSPHQGQQTTVSPHQGQQTTVSPHQGQQTTVSPHQGQQTTVSPHQGQQTTVSPHPGQQTTVSPHQGQQTTVSPHPGQQTTVSPHQGQQTTVSPHQGQQTTVSPHQGQQTTVSPHQGQQTTVSPHQGQQPGEQPCGFPGQQTTVSLHHGQQSNELYYGSPYHVSVEQPSASLKVAKAQQLAAQLPAMCRLEGGGGLLASQ			

**Table 4:** List of selected *Cannabis sativa* (hemp) proteins used for bioactive peptide in silico analysis.

Protein Name or Function	Access. No in UniProt	Length (aa)	Mass (kDa)
11S seed storage protein	A0A090CXP9 · CANSA	491	55.974
Sequence: MARSSTSLLCFTLFSLLLSHACFAQIEQMPQRSQRGGQQRQQHRWQSQCQFQRLNARQPNRRVECEAGVSEYWDIQNTEDDELHCAGVETARHTIQRRGLLLPSFLNAPMMFYVIQGRGIHGAVIPGCPETFERGTSSPSSRGYRSEGASSDEQHQKVREIKEGDMVAMPAGVADWVYNNGDSPLVLIAFVDVGNQANQLDQFSRRFHLAGNPHREQKTQQQVRARSQSRSQLRRESGEQTPNGNIFSGFDTRILAESFNVDTELAHKLQNRDDMRERIVRVRGEDLQIIAPSRIQEEERRHYSRDNGLEETFCTLRLRQNIDRPSQADIFNPRGGRLNTLNNYNLPILRFLQLTAERGVLYKNGMMAPHFNLDSHSVIYVTRGSARLQVVDDNGRNVFDGELREGQIFVVPQNFAVVKKASAQGFEWIAVKTNDNAMRNPLAGKVSAMRAMPDDVLANAFQTSREQARRLKYGRDEISVFSPSSQQTRYE			
Bifunctional protein	A0A803Q1B3 · CANSA	215	21.640
Sequence: MESLVHLPRLLVAALAIFAVLITPVFGQVSTPCNASMISSFTPCMNFVTNSSSAGTSPTSDCCNALKTLTSSGMDCLCLIVTGSVPFQVPINRSLAISLPRACNMAGVPVQCKATAAPIPAPAPASFGPALSPGDSPSSGLSPTGSSIPQPVSPALSPESDTTPLLTPPTTTGGSEAPTATTGSRSVLPPSAATTLYSSSSFLLFAMGCLVMELY			
Storage protein	A0A7J6FEU0 · CANSA	399	45.680
Sequence: MANSHRSGLIKRSSDGARVVIGTIMGVIFGFFIGMSFPSVSLNKINLPSSLISSLDVAITDIHGSSISRSFEDNGPSNVPRIYVPTNPRGAELLPPGIIVSESDFYLRRLWGEPSEDLKKKPKYLMTFTVGLDQKNNIDAAAKKLSEDFQIMLFHYDDRVTEWDEFEWSKDAIHVSVRKQTKWWYAKRFLHPDIVAAYEYIFIWDEDLGVENFNGDKYIELVKKHGLEISQPGLEPNNGLTWEMTKRRGEQEVHKDAVERPGWCDNPRQPPCAAFVEIMAPVFSRKAWRCVWHMIQNDLVHGWGLDFALRRCVEPAYEKIGVVDSQWIVHQTIPSLGNQASHHQYLLLFKQKISHSTGNSEDGKAPWEGVRARCRNEWTEFQSRLNKADEEYFAHVGKG			

## Data Availability

Data are contained within the article and Supplementary Materials.

## Supplementary Materials

The following supporting information can be downloaded at: https://www.mdpi.com/article/10.3390/molecules30183676/s1.

## Abbreviations

The following abbreviations are used in this manuscript:

ACE	Angiotensin-converting enzyme
ANOVA	Analysis of variance
BIOPEP-UWM	Bioactive peptide database of University of Warmia and Mazury in Olsztyn (UWM)
DHt	Theoretical degree of hydrolysis of protein
DPP-IV	Dipeptidyl peptidase-IV, an enzyme that plays a key role in glucose metabolism and is an important target in the treatment of type 2 diabetes
DPPH	2,2-diphenyl-1-picrylhydrazyl, a stable radical used in biochemistry to study the antioxidant properties of various substances
EBC	European Brewery Convention, a scale used to determine the color of malt and beer
FAN	Free amino nitrogen
Hep3B	The name of a human hepatocellular carcinoma (HCC) cell line that is extensively used in biomedical research
HMG-CoA reductase	Reductase of 3-hydroxy-3-methylglutaryl-coenzyme A, an enzyme that converts HMG-CoA to mevalonate, which is the controlling (limiting) step in cholesterol synthesis
HPSEC	High-performance size-exclusion chromatography
HSD	Honest significant difference
HSPs	Hemp seed polysaccharides
IL-	IL-10 (interleukin 10), IL-4 (interleukin 4), etc., which are cytokines or signaling proteins that play a key role in regulating the immune system
LC-MS/MS	Liquid chromatography–tandem mass spectrometry
MW	Molecular weight
PVPP	Polyvinylpolypyrrolidone
SEC-HPLC	Size-exclusion chromatography–high-performance liquid chromatography
TNF-α	Tumor necrosis factor alpha, is a key pro-inflammatory cytokine, which is a signaling protein produced primarily by immune system cells such as macrophages
UniProt	Universal Protein Resource, a universal protein database
