# Supplementary material for: Application of HPSEC Technique and In Silico Analysis in the Evaluation of Bioactive Peptides and Polysaccharide Profile in Wort Supplemented with Malted and Unmalted Hemp Seeds
Source: Molecules. 2025 Sep 10;30(18):3676. doi: 10.3390/molecules30183676 (PMC12472976; doi:10.3390/molecules30183676)

**Sample chromatogram from HPSEC analysis of the polypeptide fractions of barley wort with the addition of 30% unmalted hemp seeds on TSK-Gel G2000SWXl column.**

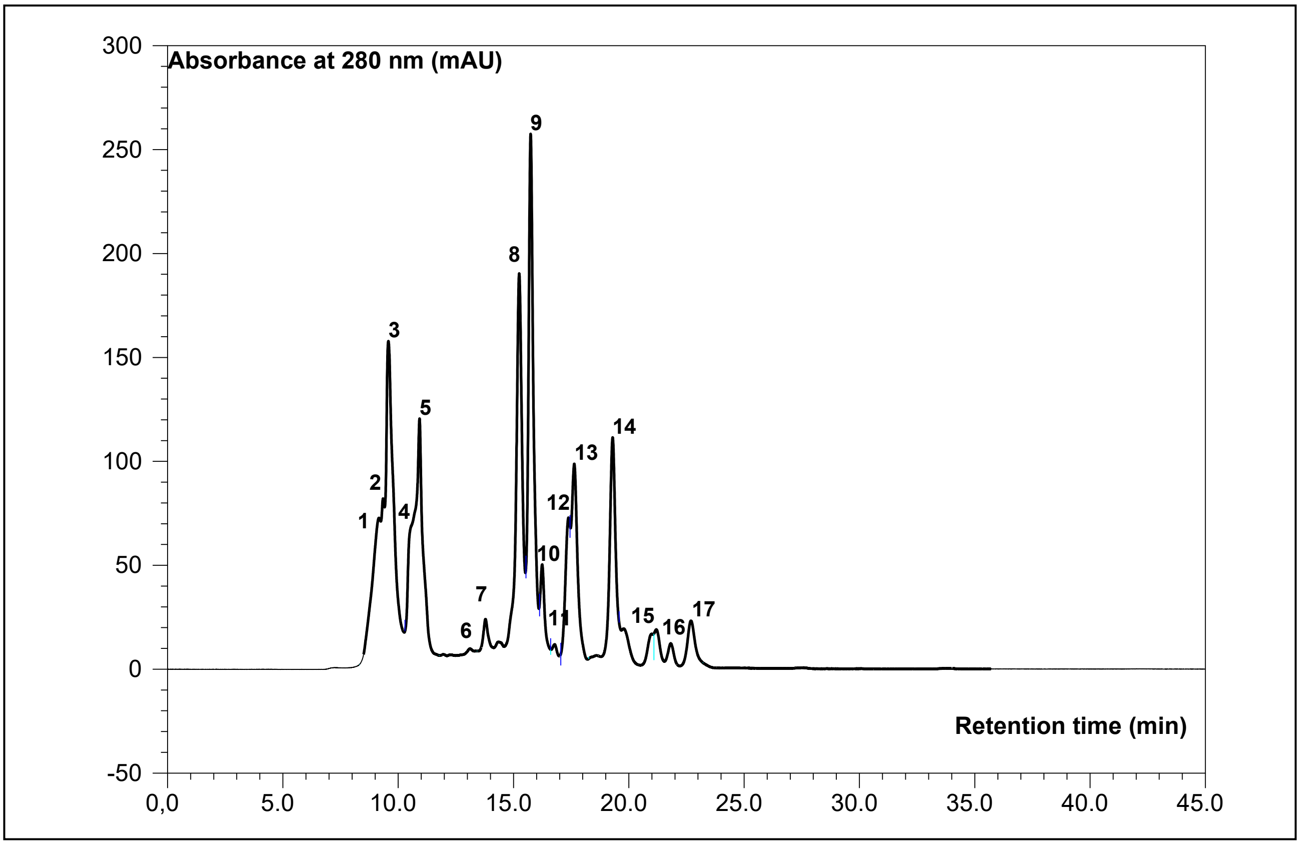

Supplement: Supplementary file 1 [file molecules-30-03676-s001.zip › molecules-3855482-supplementary.pdf]
